# Using Paulo Freire’s philosophy to share power: early insights from planning for the co-production of knowledge in research

**DOI:** 10.1186/s40900-026-00946-w

**Published:** 2026-08-13

**Authors:** Jhulia dos Santos, Mary Mbuo, Elizabeth B. Brickley, Ana Paula Lopes de Melo, Sandra Valongueiro, Loveday Penn-Kekana

**Affiliations:** 1 Department of Infectious Disease Epidemiology & International Health, London School of Hygiene & Tropical Medicine, London, WC1E 7HT UK; 2 University of Southampton, Southampton, UK; 3 Federal University of Pernambuco (UFPE), Recife, Brazil

**Keywords:** Co-production, Participatory research, Community-based participatory research, Power sharing, Empowerment, Conscientisation, Critical pedagogy, Paulo Freire, Community engagement, Global health, LMIC, Zika virus, Microcephaly.

## Abstract

**Background:**

In recent decades, there has been a growing consensus that incorporating patient, carer, community and public participation is important. How this is accomplished remains more contested. Co-production of knowledge in research is described as one potential approach; however, its theory and practice often remain unclear. The requirement of sharing power disrupts traditional research dynamics and can be challenging in many settings. Global South epistemologies, which can be interpreted as offering a framework for understanding power sharing and empowerment, hold the potential to aid co-production planning.

**Objective:**

To present a reflective, theory-informed case, drawing on preparatory efforts for the development of a project within the context of a larger research study on Congenital Zika in Brazil.

**Methods:**

By interpreting Paulo Freire’s philosophy and its concepts of dialogical action and conscientisation (critical consciousness) as a framework for understanding issues related to power, reflective planning for future co-production work with community members and groups was carried out.

**Results: early insights from our reflective planning:**

Reflective planning for co-production through a Freirean lens provided early insights on power sharing and practical challenges. Such early insights include that knowledge production is an inherently political process, requiring reflexivity and openness to tension, and the need for attention to structural power differences within research teams and between institutions. Sharing power can be an inherently challenging process due to complex power hierarchies between and within countries and current research funding and publishing dynamics.

**Conclusion:**

Early insights for our reflective planning for future co-production suggest that engagement with Freire’s philosophy at this stage offers a useful framework for critical and contextually grounded approach to co-production which re-centres power sharing, dialogue, and social transformation. Embedding such theory in planning holds the potential to lead to co-produced research which is more authentic, especially in Global South settings characterised by deep social and historical inequities.

## Abstract (significance)

This paper looks at how the ideas of Brazilian educator Paulo Freire might help researchers and communities share power more fairly when they work together to create knowledge. We draw on their experience in the LIFE Zika study in Brazil, which works with mothers and carers of children affected by Congenital Zika Syndrome to understand the impacts the Zika Virus epidemic in their lives over time. In research, the term co-production means that community members and researchers are equal partners in deciding what to study and how to do it. However, this is often hard to achieve because existing systems and power differences give researchers more control. Paulo Freire’s philosophy has the potential to help us to have a better understanding of how to share power and challenge these imbalances. Freire’s ideas encourage both researchers and communities to learn from each other and to question social inequalities. We have engaged with Freire’s ideas to prepare ourselves for upcoming co-production activities with community members. Using this theory to understand power sharing and empowerment led to initial learnings for our future co-production work, which suggest that this reflective exercise, informed by Freire’s ideas, can be beneficial for planning co-production. In this paper we share lessons learned: knowledge-making is a political process; real empowerment takes time; mutual learning and openness to conflict build stronger partnerships; and awareness of historical and global inequalities is essential. Our reflective planning suggest that Freire’s ideas can help turn co-production from a simple research method into a transformative process that promotes fairness, shared understanding, and lasting change—especially in settings marked by inequality and colonial histories.

## Introduction

In the last two decades, there has been a growing consensus that both the quality and policy relevance of most health research could be improved by incorporating higher levels of patient, carer, community or public participation [1–3]. A wide range of terminology has been used in different contexts to describe these processes and, in recent years, language describing the “‘co-production of knowledge’ has been increasingly used. There is, consequently, an emergent body of literature concerned with reporting and theorising ‘co-produced’ research [1–9].

Despite widespread support for the potential of co-production in health sciences research, ‘it is not always clear what counts as or what is meant by ‘co-production’, what it entails in practice, or what it is that is being co-produced’ [10].

The literature on co-production in health research recognises that operationalising participation and power sharing principles of co-production can be a challenging task, and consensus on how this is to be accomplished has yet to be established [11]. Theoretical and practical discussions of co-production, recommendations on how power should or could be shared, and the implications, especially when conflict or tensions develop, are not always well articulated.

Co-produced knowledge in research is meant to result from processes that by necessity reorient the relationship between researchers and participants, with participants shifting from being sources of data into a more active role in, for instance, the design of research questions or the planning of outputs [12]. Addressing power-based challenges is important, given that co-production, as commonly articulated in the literature, aims to intentionally shift power from conventional health service providers to health service users [13]. To empower historically disfranchised groups and to ultimately achieve greater equity between providers and service users is what co-production of research hopes to promote in settings where similar tensions exist between the researching and the researched [14].

What often goes insufficiently documented in the co-production of knowledge literature are the shifts in power which are inherently disruptive to the relational status quo, and which require significant effort to achieve [15, 16]. Discussing power often surfaces underlying tensions, and efforts to share power more equitably and, in some cases, for researchers to accept a rebalancing of power is necessary for success. Small, incremental steps towards the ‘promised land’ of co-production are typically a a more palatable and comfortable approach for many but remains an approach which often yields smaller shifts in power rather than transformative change.

Given the persisting scenario of limited clarity surrounding co-production in health research and its inherent challenge of power sharing, translating the enthusiasm for co-production into practice requires situated reflective planning prior to future implementation of co-production.

This paper presents a reflective, theory-informed case drawing on preparatory work for co-production workshops with community members in a sub-section of a larger research project, to develop guidelines for research going forward. From our perspectives as academics, we describe the planning which generated early insights for our future work yet to take place. This reflective planning is grounded in a Global South epistemology (Freirean theory) and in our example of preparing for co-production within a research project on the long-term impacts of the Zika epidemic in Northeast Brazil.

We define ‘co-production’ as research processes, methods or approach ‘’with a core ethos of power sharing with communities to promote mutual learning, benefits and power between researchers and participants’ [12]. We regard such processes as the enactment of a ‘philosophy’ of co-production, which announces the need for a repositioning of the typical roles of communities and researchers through power sharing and by uplifting under-valued knowledge.

We are not planning to implement co-production within the main body of the The Long-term Impacts for Families affected by the Epidemic of Zika (LIFE Zika) study (i.e. for clinical research and data linkage processes), but rather co-production workshops due to start in the future. At the time of submission, we have carried out reflective planning through the lens of Freirean philosophy for future co-production work.

Importantly, at the start of our planning for future co-production work in Brazil, we had to grapple with an apparent conceptual tension around using the ‘co-production’ terminology in our study, which is being carried out through a Global North-South collaboration (UK and Brazil).

When we presented our ideas in discussions with Brazilian academics, an important concern was part of the feedback: could ‘co-production’, as a labelled framework in the UK or Global North discourse, be yet another manifestation of epistemic coloniality and of foreign gaze [17] of a high-income country institution (LSHTM) devaluing the expertise and practices of Low- and Middle-Income Countries and Global South research teams?

The encounter with this critique prompted JdS and LPK to reflect on this issue, the ‘conceptual tension’, and on the question of which are the most appropriate theories to support co-production given that this endeavour acquires additional texture in the diverse contexts of research in the Global South. This led to ongoing discussions with others in the study team (APLdM, SV, EBB).

We understood that how we had initially articulated our future plans for co-production focused on discourse drawing from Global North references, which lacked engagement with a long tradition of participation grounded in the Global South. Upon a careful and closer look, co-production of knowledge evoked for us an approach which reflected principles aligned with Latin American ‘Popular Education’ movements, especially the work of Brazilian educator Paulo Freire.

Therefore, our planning co-production has led us to recognise the importance of grounding our work in the theoretical traditions of critical community engagement [18] which have originated in the Global South. Localising our theoretical approach, rather than imposing expectations from the Global North, has been a strategy to improve the acceptability and feasibility of our efforts especially given the extensive traditions and theoretical and methodological frameworks of community participation in many Global South research settings [18]. Central to our approach has been Paulo Freire’s philosophy of ‘emancipatory pedagogy’ [19].

The choice for Freirean theory is justified by two key elements: firstly, the development of his theoretical and methodological framework was shaped by the Brazilian and other Global South sociopolitical and cultural contexts, where our research work is located; secondly, Freire is notably an important inspiration and foundation for the development of participatory research, and his methodology adopted in community-based and action-research traditions [13, 20].

We interpret Freire’s work as offering a framework for understanding power, power sharing and empowerment in co-production. While we acknowledge that Freire didn’t specifically discuss co-production or empowerment in such terms or extensively, we argue that his philosophy can be used to support the development of a comprehensive view of empowerment, a concept which has significant relevance for thinking about co-production.

In this paper, we initially outline the arguments for co-production and challenges that have been faced implementing it as highlighted in the literature. Then, we introduce Freirean theory and describe how we believe that it holds the potential to help address the challenges of co-production, especially power sharing based on the existing literature and on our reflective planning for co-production.

## Co-production of knowledge and the issue of sharing power

The notion of ‘co-production’ originates from the recognition that the delivery of public services can benefit from ‘horizontal’ partnerships between the public sector and the communities it aims to serve [12]. In the health research field, adopting a co-production approach has been employed to address criticisms of conventional research processes, said to still reproduce ‘extractive and oppressive practices of the past’ [21], while building on work in the field of Participatory Action Research (PAR) [22, 23]. While this approach can be employed alongside Patient and Public Involvement and Engagement (PPIE) strategies, the co-production of health research presents distinctive characteristics from the more general PPIE activities. It is placed as higher in ‘ladder of participation’ models, as power is sought to be further democratised in comparison to other forms of participation.

Considered an innovative way of doing research, co-production is associated with challenges which include but are not restricted to: time needed to build relationships and make decisions as a collective, management of emotional labour, lack of integration into organisational structures and adequate funding [2, 24]. A critical understanding of participatory approaches can also reveal further challenges to the realisatation of authentic co-production, such as the unintentional constraints imposed by current conceptions of collaboration on the enactment of the ‘subjectivities and forms of knowledge’ brought on by public participants, highlighting tendencies to privilege ‘disembodied’ forms of involvement [16, 25, 26].

Having power in research, whether described explicitly or not, can be interpreted to include, according to Lokot et al., those ‘who often direct the scope of the research, hold the budget and lead the analysis’ [12]. The practice of sharing power is critical in co-production, but as power hierarchies are constructed socially, they can be reinforced by rigid processes and timeframes set by research institutions and funding bodies, constituting a challenge for researchers and communities [12].

While discussing ‘the dark side of co-production,’ and more specifically the challenges this approach, Oliver et al. highlight that many of the points of tension and costs are primarily about power [27]. As widely acknowledged in co-production literature, democratising power (and knowledge) is a complex task, especially in societal contexts where power differences, reflecting systems of oppression (e.g., based on class, education, race, gender, or age), are stark and longstanding. To avoid co-production being an ‘untenable promise’ [14], greater conceptual and theoretical coherence on participation is needed [28]. Yet, explicit naming and use of theory in co-production is recognised as limited [14, 28, 29].

Messiha et al. highlight that theories of empowerment have relevance for the co-creation of public health research, a term often linked to co-production [29]. Furthermore, the authors argue that the use of explicit theory (i.e., encompassing a set of concepts, definitions, and hypotheses) can support the advancement of co-creation, offering further transparency, consistency and rigour in the research process, potentially avoiding the risk of knowledge fragmentation and concept stretching [29].

In recent years, while funders and researchers have mobilised to promote and employ co-production, the increase in popularity may have also resulted in the term being used more loosely. This can lead to neglect of key aspects of co-production, such as addressing power dynamics. As mentioned by Lokot and Wake [12] for some scholars, the mere presence of a partnership between researchers and relevant stakeholders does not signify co-production.

### Paulo Freire’s philosophy

Brazilian educator, intellectual and activist Paulo Freire (1921–1997) is widely recognised as a scholar of global influence, whose philosophy and methodological framework has been used as a tool to challenge hegemonic conceptions of education and knowledge production through an ‘Emancipatory Pedagogy’ [19].

Freire’s legacy is linked with the idea or concept of ‘Popular Education’ -- an existing tradition within health education experiences worldwide, but especially in Brazil and Latin America, that signifies an education performed alongside the people, often the oppressed or ‘popular’ classes [13, 19].

The conception of an Emancipatory Pedagogy proposed by Freire is oriented towards societal transformation, using the concrete or lived context of those engaged in the learning process as the starting point. For the author, an emancipatory education presupposes ‘epistemological curiosity’, rigour, creativity and dialogue in this process [19].

Freire’s philosophy is inseparable from the wider context of its inception. The socio-political developments of 20th century Brazil and Latin America, where Freire lived, were the early foundations for the development of his practice and theory. This context, where the rise of popular movements and their demands to catalyse redistribution of power and the abolition of inequalities (e.g., for land reform, aiming to redistribute resources more fairly to the marginalised majority, were often met with repression from the dominant ruling classes [30].

The popularisation of ideas such as Freire’s, which proposed a radical transformation of Brazilian society, was followed by censorship, and a large number of political prisoners and exiles as a result of official State policies implemented by a civil-military dictatorship lasting 21 years [31]. Despite this, Freire’s ideas were not completely erased: they travelled from literacy campaigns and social movements to academic research, though they were at times sought to be co-opted or made invisible [32].

While the use of the term ‘pedagogy’ commonly evokes an association with formal learning spaces, Freire’s philosophy has both origins and applications which were not restricted to conventional educational settings, including experiences promoting adult learning, in schools and peasants’ associations [19].

### Freirean philosophy, power and knowledge

Freire’s philosophy and body of work is concerned with the creation of hybrid knowledge through a synthesis of different types of knowledge, much like descriptions of co-production in research. Freirean philosophy is articulated through different key concepts and ideas that produce a broader conception of knowledge production as a social, political, and collective act, supporting a deeply transformational approach to knowledge creation [33].

Freire denounces how hierarchies of knowledge are constructed within and beyond educational spaces, notably through his conception of a ‘banking model of education’, understood as hegemonic practices of education which have an inherent function of upholding the status quo and oppressive systems, and where the roles and hierarchies between educators (those ‘depositing’ content) and students (those ‘receiving’ the content).

The creation of hybrid knowledge [16] through the disruption of acknowledged hierarchies can only be actualised in an authentic manner by what Freire theorises as dialogical action. The dialogical action between subjects is understood as a horizontal relationship in search of knowledge, rather than a self-serving process, resulting in true collaboration and cultural synthesis [34]. According to Freire, in his book the ‘Pedagogy of the Oppressed’, ‘knowledge emerges only through invention and re-invention, through the restless, impatient, continuing, hopeful inquiry human beings pursue in the world, with the world, and with each other’ (p. 72) [32].

This creates the possibility that different communities, each with distinct cultural expressions, can truly begin to know each other, and promotes both the deconstruction and reconstruction of new knowledge without subjugation [33]. In this process, while Freire centers dialogue and positions education as an act of love, Góes argues that such philosophy does not shy away from recognising that conflicts, contradictions and tensions can naturally arise in the dialogical action and in relationships and cannot be readily eliminated [35]. Hybrid knowledge, albeit forged through tensions and conflicts, is positioned as the desired outcome where people with different identities and possessing different types of knowledge (i.e., researchers and communities) engage to co-produce research [29, 36].

Freire’s contributions to critical discussions of knowledge production are expressed through his concept of ‘conscientisation’: the process where, collectively, people critically develop their own consciousness. Freire described different levels of conscientisation, from magical to naïve to critical, which encompasses externally imposed sociopolitical and economic structures and processes and internalised beliefs [33, 36]. The transformation proposed by Freire in the process of conscientisation presupposes reflexivity and is described as an important principle for the co-production of research [15]. Conscientisation and dialogical action, therefore, have a dialectical relationship: the former is both required and developed by the latter [32].

The realisation of internal power and capacity to enact change and produce knowledge (i.e., conscientisation) results in shifts in identity: how we see ourselves, how we see others and therefore how we present ourselves in dialogical spaces.

While Freire himself did not outline an explicit theory of empowerment, ideas around ‘empowerment’ appear in his later work. Roso and Romanini [37] argue that although Freire did not widely use the term empowerment (either in Portuguese or English), he didn’t rule out the term’s usefulness in dialogue with scholar Ira Shor [38]. In this debate he warned that the idea of empowerment can be turned into an individualistic approach of ‘self-liberation’ in the United States’ context, and this approach must be abandoned [38].

As an alternative, with collective processes of changes in mind, Freire theorises that empowerment of specific individuals and communities must extend to the empowerment of others through a global transformation of society, constituting it as a social and political act [38, 39] which takes place through dialogical action and conscientisation. For Guareschi, another scholar of Freirean philosophy, it is possible to affirm that empowerment is intimately connected to Freire’s idea of conscientisation. Notably, dissemination of Freire’s works from the original Portuguese word *conscientização* (conscientisation) have featured translations of this concept as empowerment in English [37].

Ultimately, within Freirean philosophy, the idea of empowerment is closely linked to conscientisation and dialogical action, two key concepts within his body of work. In light of the longstanding discussions about power, sharing power and empowerment and of the need for theoretical grounding for co-production in research, Freire’s philosophy, including his conception of conscientisation, offers a framework for understanding issues around power, and suggests value for aiding the planning for co-production.

### Examples of work that has used Freirean approaches in research processes

Freire’s theory can be found across different areas of health research using participatory approaches. Reports of research work that describe engagement with Freire’s theory encompass different approaches self-described as: community-based participatory research (CBPR), action-research (AR) [13], PAR with a qualitative approach based of Freire’s pedagogy of ‘Culture Circles’ (i.e., defined as ‘dynamic spaces of learning and knowledge exchange that value the group experience and promote its participation in the construction of collective, contextualised knowledge that is committed to the transformation of reality’ [40]. Freirean informed PAR [33] ,Freirean praxis articulated with qualitative research [41], and qualitative research linked to Freire’s theoretical and methodological framework [42, 43].

We note that examples range from ‘shy engagement,’ which solely references Freire’s ideas, to operationalized approaches, which embed his theoretical and methodological framework in study design and data collection. Shy engagement with Freire’s theory is seen, for instance, in a PAR Toolkit, where authors link the learning aspect of PAR to consciousness of one’s situation as ‘a prerequisite to taking action’ and that, in this context, becoming a reflective learner with ‘conscious testing and modification of knowledge, and assuming responsibility for choices made on the basis of that knowledge’ as ideas articulated by Stuttaford and Coe [44] ,who themselves explicitly draw on Freire’s idea of conscientisation in their own work [45].

Rather than implying this to be an intentional erasure of Freire’s ideas (as it would not be surprising that authors within the field of PAR would be more likely to engage with literature which has already translated Freire’s ideas to the research context, rather than the educational context referenced in the source materials), we argue that indirect versus direct engagement with Freire’s theory might have different outcomes for participatory research practice. For instance, indirect engagement, even where ‘reflective learning’ is encouraged, and where critical reflection or thinking is mentioned, vague references to ‘training/coaching and upskilling of community researchers’ might still risk reproducing normative models in PAR and other participatory approaches, including co-production. In the context of toolkits and guidance, systematic and explicit engagement with Freire’s theory may enable more authentic co-production and PAR [22].

Another example of more comprehensive engagement with Freirean theory is the work of Wallerstein et al., which engages ‘indigenous social actors’ in Brazil. They built Freirean dialogue as part of their research methodology, by producing ‘talking maps’, using photovoice and producing a community newspaper informed by Freire’s theory-practice while also using non-participatory survey methods [13]. Similarly, an experience of using Freirean informed PAR with an Action Group (AG) of people living with dementia in Canada is reported by O’Connor et al. [33]. According to the authors, AG members were involved in all facets of the research study, including deciding output structure, focus and involvement in the development of the project’s main outcome, ‘The Flipping Stigma on its Ear Toolkit’, and taking part in integrated knowledge translation activities, such as workshops and webinar presentations. Drawing on Freirean principles, supported by group facilitators, the AG led a collective exploration of their personal experiences, linking them to broader societal responses, which authors define as an essential step for the Freirean sense of empowerment through conscientisation. This experience of the longer-term development of the AG, informed by Freire’s theory, contrasts with other accounts of Freirean informed participatory approaches which described that the substantial engagement of community members or research participants was carried out only during the data collection stage [13, 40–42, 46].

We note that other examples featured in the literature described ‘Freire inspired approaches or adapted versions of the Freirean methodologies such as ‘culture circles’’. For instance, Monteiro et al. report on research work carried out with adolescents, with the aim of empowerment and collective knowledge construction on the prevention of violence, and where one Culture Circle meeting took place in a school located in a low-income community in Recife, Pernambuco, Brazil, the adolescents were not engaged as formal ‘co-researchers’ with the incorporation of creative methods [40]. This approach contrasts with research initiatives guided by Freire’s theoretical and methodological framework, which emphasised their long-term nature, where the participatory activities took place over years and allowed for dialogue and empowerment, through a Freirean approach, to develop over time [13, 33].

Notably, Monteiro et al. listed that the study was ’involving young people from vulnerable communities, with limited access to educational resources and public policy actions and relatives with low education levels and purchasing power’ under ‘study limitations’ [40]. While the phrasing featured in the paper might not fully reflect the idea the authors wanted to convey, positioning the oppressed condition of the culture circles’ participants as a limitation, while failing to acknowledge the potential for transformation that they hold as a collective, is arguably unaligned with Freire’s ideas. This exemplifies the contradictions that might be present in such research experiences, where researchers might substantially draw on Freirean terminology or methods while also neglecting to develop their own critical consciousness to disengage with stereotypes regarding the oppressed.

On the contradictions of employing Freire’s methodology in the context of healthcare/health research, Heidemann et al. reflect on their experience and the ‘theoretical limits’ for implementation, including the deconstruction of the authoritarian relationships given the power conferred by professional knowledge on health versus lay or experiential knowledge of health users in the context of a basic health unit in São Paulo, Brazil [46]. Further challenges are highlighted such as difficulties in systematising and organising the Culture Circles, establishing dates and location for meetings and getting health workers and users to take part in a dialogical methodology aimed at reflecting on their realities.

Furthermore, such challenges can also relate to the fact that unveiling realities in a dialogical manner is inherently difficult where ‘banking models of education’, as described by Freire, or of health and research-related knowledge are still hegemonic. Heidemann et al. found that a dialogical methodology was unknown by those whom they wished to engage in the research process, requiring previous contact with participants before the start of Culture Circles [34, 42].The authors highlight the ‘anti-dialogical’ attitudes of health professionals, impairing critical thinking and that they have observed that health professionals ‘protect themselves, have difficulties in being in dialogue and working as a group in the Culture Circles, and with their authoritarian dialogue, oppress and impair health user’s thinking, resulting in a mode of thinking that is ‘shy’ and ‘restricted’. Health workers also resisted the idea of joining the meetings and reported a lack of time and excess of work [42].

O’Connor et al. highlighted ’tensions that emerged as we reflected upon these (Freire’s) ideas and their application’ [33]. The authors go on to argue that while Freire’s assertion on change being led by those oppressed is a critical core guiding value of his work, the reality of trying to implement this mandate, within the patriarchal structure in which academic research funding generally occurs, and where randomized control research is idealized as the gold standard, is extremely challenging [33].

Additional challenges of adopting Freire’s theory as a basis for co-production, conceptualizing and practice are that it can lead to the intensification of the disparities between what is achieved and what funders expect of co-production. For instance, following a Freirean approach is likely to indicate that more time is needed to co-produce [47].

While there is potential in adopting Freirean theory as a basis for co-production work, as a praxis rather than a method expanding and widening co-production, there might be significant differences between the values attributed to co-production by funders and institutions, and those attached by the participants of the co-production process, which might not always be equal or similar [33].

O’Connor et al. also reported needing to ‘walk the tightrope of providing sufficient details’ for reviewers of proposals and the ethics committee ‘while leaving the plan flexible enough to be responsive’ and aligned with their decision to draw on PAR principles. The authors found that using Freirean theory to inform research steps was only made possible by adopting a ‘tentative design’, including setting up the Action Group as the core of the project, which was outlined by researchers, while ’the actual problem and specific outcomes were left vague and undefined’ [33].

The ’vagueness of the plan’ however was, according to the authors, one of the reasons they were given for not receving funding at the first attempt. Authors were able to eventually fund the project, acquiring approval from the university ethical review committee, by building on a previous PAR study with an Action Group of people living with dementia, where stigma and discrimination were raised as important issues [33].

### Theoretical approach in our reflective planning for co-production

Informed by Freire’s ideas, in our planning, we are approaching the co-production of knowledge in our research as important and simultaneously challenging processes that we hope can help us address some of the research ‘pitfalls’ of the past [48]. We perceive co-production as having special value in the field of Congenital Zika research, where community members have articulated critiques of research including the perceived lack of mutual benefits. Referring to the families’ participation in research, and the relationship between science and participants, one woman interviewed by Fleischer expressed that: ’it has to be good for both sides’ [44].

We recognise power sharing, within this context, as a challenging endeavour that can be best guided by using Freire’s theory as a framework to understand it, and to aid the process of mutual reflection that is dependent on knowledge sharing or exchange between communities and researchers. In this manner, we seek to shift power sharing from merely abstract concepts into more concrete ways of achieving it.

Finally, in our planning, we approach co-production as an open-ended process, one which should be adequately reported, reflected upon, studied in-depth and theorised to allow future learnings and its development.

## The LIFE Zika study: context and background

The Long-term Impacts for Families affected by the Epidemic of Zika (LIFE Zika) study is being carried out to continue to understand the clinical and socioeconomic consequences of the Zika virus epidemic in Brazil, highlighting the need to carry on Zika research beyond the acute epidemic period, and implementing lessons from the past where possible [49].

The study is a collaborative effort between partners in three Brazilian cities (Recife [Universidade Federal de Pernambuco – UFPE; Universidade de Pernambuco – UPE; Instituto Aggeu Magalhães of the Fundação Oswaldo Cruz – IAM/Fiocruz ], Salvador [Centre for Data and Knowledge Integration for Health of Fiocruz – CIDACS/Fiocruz], and Rio de Janeiro [Instituto Fernandes Figueira – IFF/Fiocruz]) and the London School of Hygiene & Tropical Medicine (LSHTM) in the UK. It brings together expertise from various established research groups across a range of disciplines, which for years have been producing robust evidence to contribute to the current understanding of Congenital Zika.

Importantly, through one of its Work Packages (WP), the LIFE Zika study is concerned with understanding the socioeconomic impact for populations already marginalised due to interconnected racial, gender, and social disparities (namely Afro-Brazilian women living in low-income communities) especially women who overwhelmingly bare the main caring responsibilities of children with disabilities that have resulted from prenatal Zika virus infection.

Additionally, the study features a cross-cutting WP with the goal of engaging affected families in scientific responses to public health emergencies, using Congenital Zika research as a case study. Within the context of the LIFE Zika study, non-academic actors including parents and carers of children with Congenital Zika are recognised, at least in theory, as being of high importance for the development, interpretation, and advancement of research. As the co-production literature identifies, co-production does not run only on the intention and acknowledgement of research actors, requiring changes in practice and adequate structural support. In practice, this means that the initial ambition to embbed a co-production approach across the whole study has proved not feasible, and an attempt has been developed to carry out a co-production sub-project as part of the wider LIFE Zika study. This scenario presents unique opportunities for the wider study, and especially for JdS’s PhD study which aims to explore the complexities of community participation and co-production of knowledge in the context of Congenital Zika research in Brazil.

Since the Zika virus outbreak in Brazil (2015–2016), there have been several diversely organised advocacy groups that are led by women and other carers of children with Congenital Zika, some making direct reference to Zika-related microcephaly. One of the most active advocacy groups, *União De Mães de Anjos* - UMA, Mothers of Angels, is based in Recife, Pernambuco State, Brazil. Founded at the epicentre of the Zika epidemic, UMA has played an important role over the last decade both locally and nationally, by forging connections with groups of families from other Brazilian states as well as health and social policy makers. Through this work, UMA has effectively advocated better conditions for children and their families, including access to essential medication, access to a housing programme, and State-sponsered financial reparations and pension. For their role in advancing the rights of families of children with Congenital Zika, UMA leadership, including President Germana Soares, have been widely recognised [50].

While there has been collaboration and statements committing to further partnership between advocacy groups and Congenital Zika researchers, there have also been tensions. Leaders of advocacy groups have used scientific conferences and social media to comment on what they perceive of the behaviour of researchers, especially in the immediate aftermath of the declaration of the Zika-related Public Health Emergency. While recognising the importance of research, advocates have emphasised how research has not always been carried out in a way that respects children and carers and that families have not always benefited directly from research findings, which can be delivered too late to address their acute needs [44, 51].

Such issues have been previously reported in the literature: during the Zika pandemic, a scientific rush in Brazil developed in the face of the need to provide answers and assistance to families [52] who were in turn ‘drafted’ to take part in research and sought out by local and international journalists [53]. For some, the initial hope in science decreased due to a kind of ‘research fatigue’, with perception that communication of research findings was insufficient and that there was an overall lack of reciprocity in benefits [44]. The Congenital Zika research community has echoed these concerns, calling attention to the extractive and colonial nature of international ‘parachute research’ [54] and to the importance of ‘centering the knowledge and expertise of local researchers, public health practitioners, and affected communities’ through locally-led, respectful research that directly informs the multidisciplinary clinical care of participating families and guides municipal, state, and national public health responses [55].

An emblematic situation took place in 2018 when an open letter, read out by the UMA advocacy leadership to the audience of the Brazilian Tropical Medicine Society meeting where Congenital Zika research findings were being discussed [51]. The content of the letter expressed issues not unique to one research group or to Congenital Zika research. It raised concerns that mothers and carers were sometimes feeling used and dismissed as not being educated enough to understand research, calling for ‘nothing about us without us’, a phrase often used in the disability community. They then went on to call for closer engagement of research with affected families, stating ‘We want to be partners, to collaborate, to contribute, to be part of the process, and to understand the discoveries so that everyone can move forward hand in hand (*Queremos ser parceiras*,* colaborar*,* contribuir*,* fazer parte da construção*,* entender as descobertas para que todos andem de mãos dadas*)’ [51]. The message was met with a response-letter from the event’s organisers, which deemed the women’s intervention in the scientific meeting as having an unnecessarily aggressive tone, but that also apologised for the lack of representation of the advocacy groups at the meeting and emphasised ‘sincere respect to the mission of UMA and all the Social Movements that are with us in partnership in the fight against infectious and neglected diseases (*sincero respeito à missão da UMA e de todos os Movimentos Sociais que estão junto conosco em parceria no enfrentamento de doenças infecciosas e negligenciadas*)’ [56].

Such tensions have not disappeared and can be understood as resulting from a combination of causes. From the advocacy groups’ perspective, women and carers have reported feeling empowered as the protagonists in the fight for the rights of their children including in obtaining social grants but relatively sidelined in the realm of scientific research. Congenital Zika researchers have, in turn, countered that some of the frustrations of the advocacy groups may reflect an inherent limitation of research to produce direct benefits for studied populations, as some of the findings (e.g., the causal link of Zika virus and microcephaly) will mainly be relevant to protecting the health of future populations. Another challenge can be a misalignment in expectations or unclear communication of roles in relation to scientific research. For example, research groups may, at times, underestimate the specific medical knowledge of carers, while carers may, at times, overestimate researchers’ ability to drive public health policy-making decisions or misunderstand the constraints of clinical research funding. The aftermath of the Zika epidemic illustrates how relationships between actors across advocacy and research groups have ranged from paternalism to a search for authentic collaboration.

Within the LIFE Zika Study, recognising the diverse ways in which the principles of community participation and different participatory practices in research can be interpreted and operationalised, we are planning to carry out co-production workshops, aiming to move away, and at times alongside, from one-time consultation and participation, and towards a more reflective and dialogical practice (as described by Freire), which has demanded increased time and resources. It has also demanded engagement with theory, making ideas such as ‘empowering communities’ less abstract and more tangible.

## Results: early insights from our reflective planning

In its overall study goals, the LIFE Zika study aims to produce research outcomes which are considered important by affected families through the operationalisation of WP4 (Engaging affected families as research partners) referencing the co-production of knowledge as an important approach. To this end, we have engaged with the literature which reviewed documents and communication produced by family advocacy groups in different platforms including conference discussed previously. As leads of WP4, within the LIFE Zika meetings with the wider team, across different WPs, we have promoted discussions about co-production and embedding other levels of community participation within the study. Under the umbrella of the LIFE Zika study, we are developing a sub-project to carry out the co-production of a guidance for ethical research, led by women carers of children with Congenital Zika in Recife, Pernambuco State, Brazil.

To shape the planning of the co-production work, two participatory meetings led by JdS were held in Recife in late 2023 and late 2024 in collaboration with the UMA advocacy group. Through these meetings, we further consolidated the need to co-produce ethical guidelines for long-term research with families affected by epidemics, using as a model other ethical guidelines produced by other highly researched groups, such as survivors of gender-based violence or people who use drugs [57, 58].

Guided by a Freirean approach to knowledge production, the academic team (led by JdS), in conversation with the leadership of family advocacy groups, have engaged in ongoing preparation for a series of planned co-production workshops to demystify the research, ethics process and discuss key points of intervention for ethical research. Set to take place in Recife in 2026, plans for the workshops include invitations to representatives from research and ethics committees to share experiences and knowledge in a dialogical process where the women are encouraged be the key actors of decision-making about the workshops and the content of the ethical guidelines. We anticipate challenges in promoting dialogical exchanges, and we also seek to identify opportunities.

In this process of reflective planning of the co-production workshops, we have tapped into the existing expertise held by advocacy groups’ leaders, which they have developed through their participation in public policy councils, research ethics committees, advocacy rights campaigns and other forums. This effort is informed by the Freirean conception that the prior knowledge and experiences of those participating in the knowledge production process must be brought in and examined. These encounters could also provide the opportunity to democratise knowledge about research within and beyond advocacy groups, where power dynamics are expected to exist as in other organisations.

We have also reflected on the need for the inclusion of lived experience reflecting participants’ sociocultural contexts within our academic team – and the person with this experience (JdS) has been central to the planning of the future co-production workshops, as well as for advancing Freire’s dialogical approach within the research process. JdS holds the experience of growing up and living in a low-income community in Recife, an experience that is shared by most of LIFE Zika’s research participants but not by the majority of the research team. This lived experience, beyond the simplifying label of ‘being from a disadvantaged background’ can be best described as one of belonging to the ‘periphery’ or a *favela*, shared with many other Afro-Brazilian and working-class communities.

While lived experience of a community’s context among academics can be described as an asset for authentic community participation, it can also come with great costs. In our project and in the efforts to make co-production happen, this has also demanded considerable emotional labour from the researcher with lived experience, who has faced considerable push back and debate over some of what she has proposed, and substantial support from some more experienced team members, as academia is still widely considered a constraining environment for individuals from marginalised groups and especially for work that aim to disrupt existing power hierarchies.

Engagement with Freire’s theory has provided us with informative early insights for how to operationalise future co-production processes in our work. This theory-informed reflective planning has highlighted challenges related to time, funding, and ethics review processes, as well as limitations in reflexivity among those holding power, within the context of a larger study conducted in a country (Brazil) with entrenched social and economic inequalities, and through partnership with a Global North institution (LSHTM, in the UK, where the PhD is based) that has faced sustained critique regarding its ongoing attempts to address its colonial legacy [59].

Our conception of co-production attempts to uphold the principles of power sharing by, from the planning stage, embracing, reflecting and discussing tensions. We also seek, by embodying Freirean philosophy, to be critical of the literature and our own positionalities and actions, and to action power sharing through mutual knowledge exchange, articulating Freirean concepts such as conscientisation and dialogical action.

To further reflection beyond the planning stage, the upcoming co-production workshops are planned to be the object of study of JdS’s PhD study on understanding the complexities of co-production of knowledge, through participant observation and follow-up interviews.

These early insights from our reflective planning for the co-production of ethical guidelines for research, led by women and carers of children with Congenital Zika Syndrome, can be summarised in the following key points, which could provide learning for research work in LMICs contexts and beyond.

### Knowledge (co-)production is an inherently political endeavour

Knowledge, according to Freire, is never neutral [34]. An important takeaway of Freire’s theory for co-production in research is the need to actively and consistently understand knowledge (co-)production as inherently political, rather than neutral [7]. Using theory has enabled us to repoliticise co-production in our planning.

Freire’s philosophy has and can continue to guide us in a sustained effort to ‘read the world’ and the reality where our work develops, unveiling myths which exist within society and that might prevail within the practice our research teams [34]. One such myths is that sharing power to co-produce knowledge can always be achieved solely based on ‘diversifying the seats at the table’. To only invite others to join us, or to even do one-time ‘outreach’ activities, without reflecting on our own understanding of participation, or power dynamics in these processes, is a political choice.

The dominant rhetoric found in guidelines for co-production positions power sharing as something that can be achieved through building relationships, without highlighting the need to examine the political context where co-production develops, and how it can hinder or support power sharing and empowerment. Regardless of how one chooses to guide co-production practice, a set of interests will be represented. Freire teaches us that whether you address these interests and power or not (by using a more technocratic approach), this is fundamentally a political process.

Without the recognition that knowledge production and sharing of power are inherently political endeavors that should be approached with intentionality and reflexivity, we risk misinterpreting Freire’s ideas and consequently their implementation in planning for the co-production of research.

### Authentic power sharing and empowerment can be mediated through the process of conscientisation

It is imperative for the co-production of research to address the ‘culture of silence’ [34] that keeps social and economic inequities unexamined [16]. Authentic co-production needs to not just acknowledge systems of oppression but closely to examine them in a collective manner ‘through a dialogical problem-posing method, a reciprocal questioning that fosters critical engagement with real-life situations’ [33]. Such efforts require the development of critical consciousness among researchers, who can learn to ‘read the world’ differently [60, 61].

Freirean informed co-production holds the potential to guide ‘the research process in important – and sometimes contradictory and uncomfortable – ways’ in the typical fashion of Freirean philosophy, which is recognised as a ‘living pedagogy of discomfort’ [36].

The process of conscientisation by researchers is likely to require extensive time and effort, going beyond the ‘good intentions’ of individual researchers, and should be adequately supported by research teams, their institutions and funders. Critically reflection on the experience of co-producing research, including listening to how communities perceive their own participation, should be embedded in the research process. This is proving to be challenging, at the planning stage for co-production, within this research project where both carers and researchers have busy timetables and many conflicting responsibilities. More and better reporting of the methodology and experience of co-production will better prepare researchers to do this type of valuable work, which is often challenging and may evoke tensions.

### Mutual exchange of knowledge, capacity building and development of personal skills are essential values for authentic co-production

Where co-production and overall *meaningful* community participation are to be achieved (i.e., in contrast to tokenistic community participation), it is neither sufficient or efficient to ask communities only for their input/opinion as a ‘one off’ activity. It is preferable to facilitate the knowledge and tools in a dialogical manner, continually exchanging with and learning from communities.

Capacity building and the development of ‘personal skills to challenge disempowering relations’, which might only be developed in a longer timeline across years, might increase the impact of the co-production outputs, especially when participants can be active in publicising and further developing the work [26]. Our experience suggests that engagement with Freirean theory during the planning for co-production provides an opportunity for researchers to think of co-production outputs beyond normative research ‘deliverables’, especially in scenarios where capacity building is understood as an important outcome of research.

### As researchers, it is key to not just promote empowerment but to actively challenge understandings and attitudes that can be disempowering

As co-production might be happening alongside other forms of community engagement (e.g., consultation) in the same study, it is important to remain aware of how it may be uncomfortable to create one relational dynamic in one component of the study, when different power dynamics between researchers and participants may persist in other areas of the same study.

Disempowering attitudes by researchers, however unintentional, can translate into paternalistic practices. For example, researchers may retain gatekeeping authority in spaces labelled ‘collaborative’, inviting only ‘good’ participants and excluding those deemed ‘troublemakers’. When authentic power sharing is being promoted, conflict is likely to arise, as pointed out in Freirean theory as part of the dialogical process which can be generative: researchers need to be open to critiques from communities which might have their own ideas of what constitutes authentic co-production.

Following a Freirean approach, when facing critiques from communities about our work and/or approach to participation, we must take it as an opportunity for opening up dialogical communication, rather than pushing those with challenging viewpoints away. To avoid disempowering practices, researchers should cultivate a ‘reflexive’ space to unpack their co-production practice and engage in uncomfortable learning [46].

### Co-production processes might translate into challenging normative practices which may unintentionally impair participation

Authentic co-production requires problematising normative approaches to research, and the guidelines we are expected to comply with [23]. In some instances, such as reported by O’Connor et al. study co-leads needed to challenge conventional practices of assuring anonymity ‘by inviting each action group member to determine the degree of anonymity they wished to maintain in the research process’’. In their study, which involved adults with dementia, the approach was not challenged by the university ethics board ‘possibly related to previous conversations and because rights to presumed capacity were explicitly affirmed in the submission with a plan for addressing potential cognitive changes that might influence ability to provide informed consent’ [33].

### Co-production, and its theoretical underpinnings must address power hierarchies between and within countries

Freire aimed to combat an implicit ideology of paternalism in society [34]. The application of his theory in co-production should not neglect this aspect. Even when our research institutions/universities’ values attempt to grapple with their (neo)colonial legacies and research protocols speak of promoting equality and sharing power with communities, in the reality of our research work, however good our intentions, we are faced with stark inequalities, including between research participants and researchers and within research teams.

Importantly, a shift away from paternalism towards authentic co-production requires changes in attitudes of academics, who are often under pressure from the logistic constraints of the academic environments in which they work, and which may prioritise funding and publication, rather than co-production. The challenge is that a collective process of conscientisation as described by Freire, would require a transformation of relationships with communities, funding bodies, and academia that is beyond the scope of work in any one study. More simply, in being guided by Freire’s critical pedagogy as it offers a framework to understand empowerment, one cannot call oneself ‘for’ authentic co-production, without developing a critical consciousness to embody this philosophy and to reflect on their own practices and how paternalism might still be present.

True and substantial engagement with Freire’s ideas prompts us to not look away from the reality of inequalities and paternalism within our fields, institutions and research teams, prompting us into action by developing a critical outlook on our own roles, the constraints of academia and the potential we all hold to transform our relationships with each other and advance our practices forwards. Smith et al. highlight the issue of tokenism instead of ‘true’ co-production in their work [12, 25]. We argue that considering this aspect, co-production can be interpreted dialectically, with the potential to be both promising and problematic, making a critical approach to it an imperative.

## Discussion

From our reflective planning practice in the LIFE Zika study, we argue that Freire’s philosophy is one emblematic example of how Global South theoretical and methodological traditions can aid the development of co-production of knowledge in the South and North, considering the specificities of the different contexts.

Global South epistemologies have pioneered pushing ‘Ivory Tower’ academia to engage with community realities and knowledge, constituting critical traditions of community engagement [13]. Flores et al., state that ‘connection between universities and society is not new for Latin American Universities’, with the First Latin American Conference on Community Engagement and Cultural Exchange dating from 1958 [18].While critically reviewing the history of traditions of community participation, the authors note ‘at a time (.) when some countries export accreditation systems tending to standardisation, there is a risk of forgetting the diversity of trajectories, expressions, and meanings at a global level’ [18].

Aligned with Freire’s ideas, the critical tradition of Latin American community engagement centres the commitment to the ‘subalternised sectors’ of society and the conception of engagement as a pedagogical process. In this conception, to be authentic, engagement must be ‘planned, dynamic, systemic, interdisciplinary, permanent, mandatory, and coordinated with other social sectors’. In the Latin American critical tradition, there has been special attention that community engagement should be ‘stripped of all paternalistic and patronising character’ [18].

Within the Latin American ethos discussed by Flores et al., its grounding in Freirean philosophy is expressed in the understanding put forward by the Latin American and Caribbean Union of Community Engagement (ULEU), which defines ‘the interaction and permanent learning between the university and society’ as the central axis of community engagement (termed *extensión*,* vinculación y acción social* in the original Spanish) to allow for the ‘construction of truthful, critical, and ethical communicative processes, oriented to the transformation of the reality of the region’ [18].

Freirean theoretical and methodological frameworks hold the potential to guide us to address what is still underacknowledged in mainstream co-production frameworks: sharing power does not automatically occur when ‘co-production’ is embedded in research protocols, as it is often answering funders or institutional expectations or simply by building relationships without intentional dialogue. Engagement with this theory during the planning stage of co-production work indicates the means to re-ignite the critical iterations of community engagement and knowledge (co-)production articulated and actualised in Global South traditions of critical community engagement since the 1960s [18].

Importantly, for research conducted in Global South countries, but carried out in collaboration and with funding from the North, such as in our own LIFE Zika study, reflection articulated through engagement with Freirean theory used as framework for understanding empowerment and power sharing can especially aid critical examinations of power dynamics within countries in the Global South, alongside the existing Global North-South axes of inequalities, potentially facilitating context-appropriate adaptations in strategy.

## Conclusion

This paper details how Freire’s philosophy can potentially be used to support a reflective and critical planning for future co-production work, and to share power, drawing from the experience within a research study in Brazil. Freire’s theory provides ideas, through its principles and methods, on how to promote power sharing through conscientisation, knowledge exchange, capacity building and authentic dialogue - all of which are essential to achieve co-production. Our experience suggests that planning for future co-production work can benefit from in-depth political engagement with Freire’s theory, where interpreted as a framework for understanding empowerment and to grapple with the complexity of sharing power, especially in Global South contexts where stark power differences linked to colonial histories remain relevant.

If we agree with Freire’s understandings of knowledge production and power, and the political nature of how power is constructed in the social spaces - where co-production is practiced - when planning for co-production, we need to fundamentally develop critical understandings of empowerment and power sharing.

## Data Availability

No datasets were generated or analysed during the current study.
